# New York State Veterinary Medical Society

**Published:** 1893-12

**Authors:** 


### PROCEEDINGS OF THE FOURTH SEMI-ANNUAL MEETING OF THE NEW YORK STATE VETERINARY MEDICAL SOCIETY.

(*Continued from page 361.*)

### REVISED BY-LAWS.

### CHAPTER I.

SECTION 1. This Association shall be known as the NEW YORK STATE VETERINARY MEDICAL SOCIETY.

SEC. 2. The objects of the Society are to aid in elevating and regulating the practice of veterinary medicine and surgery in this State, and to contribute to the diffusion of true science, and particularly to the knowledge of the healing art, and to unite our energies, efforts and sympathies for the mutual benefit of all its members.

SEC. 3. This Society shall consist of qualified veterinarians residing in the State of New York.

SEC. 4. The officers shall be a President, a Vice-President, a Secretary-Treasurer and five Censors, all of whom shall be elected by ballot at an annual meeting, and a majority of all the voters present shall be necessary for a choice.

They shall enter upon their duties at the close of the meeting at which they were elected, and hold their offices for the term of two years, or until their successors are chosen, to whom they shall then, without delay, deliver up and transfer all moneys, books, manuscripts, vouchers and all other property belonging to the Society in their possession.

SEC. 5. This Society shall convene once in each year, and this meeting shall be known as the Annual Meeting, which shall be called no later than the fifteenth of September and not prior to the fifth of the same month. This meeting shall continue for two days. The opening hour shall be at eleven o'clock A. M., and may adjourn on the second day at such hour as a majority of the members see fit to determine.

### CHAPTER II.

SECTION 1. It shall be the duty of the President to preside at all meetings of the Society, and to preserve order and decorum. He shall, at the opening of the Annual meeting, make a communication to the Society, setting forth the conditions of the veterinary medical profession in the State, with such suggestions in relation to its improvement as he shall deem appropriate.

SEC. 2. The Vice-President in the absence of, or at the request of the President, shall perform the duties of his office.

SEC. 3. It shall be the duty of the Secretary-Treasurer to record the minutes of the meetings of the Society. He shall keep on file all documents and certificates in relation to the Society that may be deposited with him. He shall give due notice to each member of the time and place of each meeting. He shall notify the members of the several committees of their appointment when necessary. He shall receive all applications for membership, fees and other moneys due the Society. It shall be his duty to conduct the correspondence of the Society. He shall retain copies of all letters written by him in behalf of the Society, preserve and file all letters and communications received by him in his official capacity, and he shall report the same at all meetings of the Society. For such services the Secretary shall receive one hundred dollars per annum.

SEC. 4. The Secretary-Treasurer shall keep and be accountable for all money belonging to this Society received by him, and shall thereout pay such warrants as may be drawn for the use of the Society. He shall give security, to be approved and retained by the President, for the safe keeping of said moneys, and on the first day of each Annual meeting present a detailed report of the state of the treasury.

SEC. 5. It shall be the duty of the Censors to inspect the credentials of all applicants for membership, and to entertain none who present themselves unqualified. Credentials for application must consist of:

FIRST. A diploma from a legally chartered and incorporated College or University, whose reputation as an institution teaching the art of veterinary science is acknowledged and accepted as worthy in name, the instructors of such an institution four of whom must be legally qualified veterinarians.

SECOND. Any applicant for membership shall submit his name upon one of the Society's application blanks, duly vouched for by two of the members of the Society. And the applicant shall be of good social and moral standing, and honest in all his dealing with his fellow men.

THIRD. All candidates reported favorably by the Censors shall be balloted for by the Society. Those receiving a two-thirds vote of the members present shall be declared elected; an adverse report will debar the applicant from membership.

SEC. 6. It shall also be the duties of the officers when satisfied that the applicant is entitled to membership to order the Secretary to fill out the following certificate:

SEC. 7. THIS IS TO CERTIFY, that ......................................
is a member of the New York State Veterinary Medical Society, that the above....
.............................................having produced satisfactory evidence, is entitled to full membership and the immunity of this Society.

IN TESTIMONY WHEREOF, we have affixed our hands and the seal of the Society.

This ...........................day of.......................................

.......................................President.

.......................................Secretary.

(L. S.) .......................................Censors.

SEC. 8. Every candidate, before signing the By-Laws and receiving a certificate, shall be required to pay an initiation fee of Five Dollars ($5.00), together with the annual dues, which sum shall be appropriated to the use of the Society. The dues shall be $2.00 per annum, payable in advance.

Upon the presentation of the Secretary-Treasurer's receipt for this amount the Secretary shall present the candidates with a copy of the By-Laws and certificate of membership.

SEC. 9. The President, Vice-President, Secretary-Treasurer, and Chairman of the Standing Committees shall constitute the Executive Committee, whose duty it shall be to carry into execution the By-Laws and regulations of this Society during its adjournment.

### CHAPTER III.

SECTION 1. The committees of this Society shall be Standing and Temporary Committees.

The Standing committees are those whose duties continue through the year, and they shall be appointed by the President.

Temporary committees are those whose functions cease with the meetings of the Society. They shall be appointed by the President.

SEC. 2. There shall be a Standing committee of three, of whom the Secretary-Treasurer shall be one, to be called the Committee of Arrangements, whose duty it shall be to provide convenient rooms for the meeting of the Society and to attend to the reception of the delegates from other societies, and of invited guests.

SEC. 3. There shall be a Standing committee on Legislation, of three members, including the President and Secretary-Treasurer, the remaining one to be elected by the Society, who shall watch the course of State legislation pertaining to Veterinary medicine, and to take charge of such matters pertaining to medical legislation as shall be referred to them by this Society.

SEC. 4. There shall be a Standing committee of three, of whom the Secretary-Treasurer shall be one, to be called the Committee on By-Laws. Their duties shall be to make minutes of all revisions and additions to the present code, and prepare the same for publication

SEC. 5. The Secretary-Treasurer shall have power to appoint from the Society, in case of necessity, any member to act as his assistant.

SEC 6. The President shall, after the presentation of the Secretary-Treasurer's report, appoint an Auditing Committee of three members to examine said report, compare it with the vouchers, and submit the result of their examination to the Society.

SEC. 7. Immediately after the assembling of the Society, the President shall appoint as many temporary committees as the business interests of that meeting shall warrant.

SEC. 8. These By-Laws may be altered or amended by two-thirds of the members present, provided the said alteration or amendment has been proposed in writing at a previous meeting by two members ; and any article or section of these By-Laws may be suspended during a single meeting by the unanimous consent of the members present.

SEC. 9 Any member who shall be absent from two annual meetings, without a satisfactory excuse, may have his name dropped from the roll.

SEC. 10. All members two years in arrears for dues, may have their names dropped from the roll.

### DUTIES OF MEMBERS.

It shall be the duty of each member of the Society to attend each Annual meeting of the Society, so long as he shall be a member thereof, unless he shall be necessarily detained, in which case he shall communicate with the Secretary on or before the day of the Annual meeting, giving legitimate reasons for his absence.

Each member in attendance at meetings shall contribute to the interest and intelligence of the same by taking part in the discussions of the subjects presented. or by reading some professional communication relating to his daily practice or by the presentation of a prepared thesis of a professional nature.

### Conduct of Members.

Any member who shall be deemed in the judgment of the Society to be unfit for continuing in membership. either in consequence of unprofessional action or from having adopted a course of conduct calculated to throw the Society into disrepute, shall be cited to appear at the next regular meeting and show cause why he should not be expelled. and if, at the said meeting, he refuses to appear, or if his defense should not be deemed sufficient, he may be expelled by an affirmative vote of two-thirds of the members present.

### Rules for Members.

No member should leave the assembly room during the reading of any paper, or the discussion of the same. without first obtaining permission of the President.

### The Appointing of Essayists

Before the adjournment of the Annual meeting the President shall designate five members as essayists for the next Annual meeting, and before announcing the same he shall obtain the consent of the persons so named, together (if they desire) the title of the subject they wish to discuss.

### Duties Necessary to be Eligible to the Office of the President.

No member shall be eligible to the office of President of the Society until he has been a member of the Society for two years and in good standing.

### Order of Business.

1. The Secretary shall call the roll.
2. The Secretary shall read the minutes of the previous meeting.
3. President's address.
4. Secretary's report.
5. Application for membership.
6. Board of Censor's report.
7. Election of members.
8. Report of committees.
9. Miscellaneous Business.
10. Treasurer's report.
11. Reading of papers, etc.
12. Election of officers.
13. Appointment of Committee's and Essayists.
14. Place of next meeting.
15. Adjournment.

### Reinstatement of Members.

Section 1. Any member who becomes a non-member through his delinquency to pay dues and assessments may be restored to full membership in the Society by paying to the Secretary Treasurer the full amount of his indebtedness at the time his name was stricken from the roll of members, and by the further payment of all annual dues which have accumulated since the day he was declared a non-member to the day of his restoration, which shall be left to the discretion of the Board of Censors.

Any member who has been expelled from the society for violation of its laws, may through five members in good standing petition for his reinstatement.

Said petition shall be referred to the Board of Censors, with instructions to investigate the merits of the petitioner and to report their decision in the association. If favorable, a two-thirds affirmative vote of the members present will restore him to membership, after complying with any other requirements necessary. An adverse report is final.

WHEN AN ASSESSMENT CAN BE MADE.

Whenever there shall be *bona fide* bills of the Society's obligations presented for payment, and it shall appear that the Secretary-Treasurer has no money, or not enough to redeem the same, if in the judgment of the majority of the members present it shall be deemed expedient to cancel the obligation, an equal assessment can be made of all the members sufficient to relieve the entire indebtedness, or a portion thereof. But no assessment should ever be made to exceed the sum of five dollars per capita of the membership without the unanimous consent of all the members thereof.

---

*Approved by the Society.*

### AN ACT

TO FIX THE PAY, ALLOWANCES AND RANK OF THE VETERINARIANS OF THE UNITED STATES ARMY.

Introduced in the Senate by Mr. Cameron.

Introduced in the House by Mr. Robinson.

Endorsed and recommended by Gen. Schofield and the War Department.

*BE IT ENACTED by the Senate and House of Representatives of the United States of America, in Congress assembled:*

SECTION I. That the pay of the United States Army Veterinarians shall be one hundred and twenty-five dollars ($125) per month, with the allowances, pensions, retirement, tenure of office and relative rank of a Second Lieutenant of Cavalry. *Provided*, however, that hereafter candidates for the United States Army Veterinary service shall comply with all the preliminaries now required from candidates for "The Army Medical Corps," and shall pass such examinations as the Secretary of War may direct.

SECTION II. This Act shall be in force and effect from and after its approval.

SECTION III. All Acts inconsistent with this Act are hereby repealed.

